# Partially Hydrolyzed Guar Gum Combined with a Low-Fat Diet Ameliorates Type 2 Diabetes Mellitus via Modulating Gut Microbiota and Fecal Metabolites

**DOI:** 10.3390/nu17233746

**Published:** 2025-11-28

**Authors:** Zhiqiang Cao, Hongxia Li, Quantao Cai, Li Chen, Liangzhong Liu, Yuhan Tang, Zhe Zhu, Ping Yao

**Affiliations:** 1 Department of Nutrition and Food Hygiene, School of Public Health, Tongji Medical College, Huazhong University of Science and Technology, Wuhan 430030, China; 2 Hubei Key Laboratory of Food Nutrition and Safety, School of Public Health, Tongji Medical College, Huazhong University of Science and Technology, Wuhan 430030, China; 3 Ministry of Education Key Laboratory of Environment, School of Public Health, Tongji Medical College, Huazhong University of Science and Technology, Wuhan 430030, China; 4 School of Food Science and Engineering, Wuhan Polytechnic University, Wuhan 430048, China; 5 Wuhan Longfengyuan Biotechnology Co., Ltd., Wuhan 430040, China

**Keywords:** low-fat diet, partially hydrolyzed guar gum, gut microbiota, short-chain fatty acids, type 2 diabetes mellitus

## Abstract

**Background**: Low-fat diet (LFD) is widely applied in type 2 diabetes mellitus (T2DM), but the limited efficacy and difficulty in maintaining it hinder its wider promotion. Partially hydrolyzed guar gum (PHGG) is well-known as a probiotic in modulating gut microbiota, which is crucial in T2DM. However, the combined effects of LFD and PHGG remain unknown. **Methods**: Mice with T2DM were divided into 4 groups: T2DM control (DM-high-fat diet), LFD alone (DM-LFD), or LFD combined with low or high doses of PHGG (PHGG-L/H, 2.5% and 7.5% (*w*/*w*)) for 12 weeks. Serum lipid profiles, fasting blood glucose (FBG), HOMA-IR, and intraperitoneal glucose tolerance test (IPGTT) were assessed. Furthermore, microbiota composition, fecal metabolites, and fecal short-chain fatty acids (SCFAs) were determined by 16S rRNA gene sequencing, untargeted metabolomics, and gas chromatography-mass spectrometry, respectively. **Results**: LFD improved dyslipidemia but not glucose metabolism disorders. However, PHGG remarkably decreased FBG and HOMA-IR, and increased glucose tolerance. PHGG upregulated the abundance of SCFA-producing bacteria, including the genera *Dubosiella*, *Bifidobacterium*, and *Ruminococcus*, which were negatively correlated with FBG, HOMA-IR, and AUC (IPGTT). Moreover, the metabolic pathways altered by PHGG were enriched in tryptophan, tyrosine, and galactose metabolism. Fecal propionic acid and butyric acid, positively correlated with the abundance of genera *Dubosiella* and *Ruminococcus*, were markedly decreased by 50% and 44% in the DM-LFD group, but increased 2-fold after PHGG supplementation. **Conclusions**: PHGG combined with LFD might be a potential strategy to ameliorate glucose metabolic disorders, likely through modulating gut microbiota and the production of propionic acid and butyric acid.

## 1. Introduction

A recent report from the WHO indicates that diabetes ranks as the eighth leading cause of mortality worldwide, which is considered one of the “big four” major noncommunicable diseases, alongside cardiovascular disorders, cancer, and chronic respiratory disease [1]. According to the latest statistics from the International Diabetes Federation (IDF), the global prevalence of diabetes is rising rapidly, with 537 million people (10.5%) aged 20–79 years affected in 2021, while the total number of people with diabetes is expected to rise to 643 million (11.3%) in 2030 and 783 million (12.2%) in 2045 [2]. Type 2 diabetes mellitus (T2DM), which accounts for nearly 90% of diabetes, is characterized by micro- and macrovascular complications that worsen quality of life and cause more than 5.1 million deaths worldwide each year [2,3].

The pathogenesis of T2DM is complex. As explained by the twin cycle hypothesis, it involves the interaction of self-reinforcing cycles of fat accumulation inside the liver and pancreas, driven by modest but chronic positive calorie balance [4]. The initial cycle is characterized by excessive dietary fat-induced hepatic lipid accumulation, causing liver insulin resistance, which leads to a rise in fasting blood glucose and induces excess insulin production [5]. Furthermore, hepatic fat deposition promotes lipid release into the circulation, exposing extrahepatic tissues like the pancreatic islets to lipotoxicity [6]. Thus, the synthesis and secretion of insulin may be hindered, resulting in pancreatic β-cell hypersteatosis, eventually leading to an increase in blood glucose levels [7]. Therefore, reducing lipid deposition in the liver has been well recognized as an effective strategy for T2DM remission by preventing the first vicious cycle of hyperinsulinemia and suppressing the production of hepatic glucose.

In recent years, a low-fat diet (LFD) has been widely adopted in the nutritional management of T2DM patients [8]. Studies indicate that LFD administration for 6 months could reduce liver steatosis, decrease serum triglycerides (TG), increase serum high-density lipoprotein cholesterol (HDL-C), and lower fasting blood glucose (FBG) in people with T2DM [9]. Although LFD seems to show a potential protective effect on T2DM, a low response rate has always been one of the major problems in most intervention studies [10]. For instance, the Diabetes Remission Clinical Trial (DiRECT), a randomized controlled trial of an LFD with the highest remission rate of T2DM so far, demonstrated that only 46% of participants sustained remission at 12 months, highlighting the challenge in maintaining long-term efficacy [11]. This challenge is also exemplified by the Look AHEAD study; 11.5% of participants achieved remission through a combined LFD and intensive physical activity regimen, but approximately one-third of them reverted to a clinical diabetes status within the following year [12]. Therefore, more nutritional strategies building upon the foundation of LFD are urgently needed to improve its therapeutic efficacy in T2DM management.

The data on the number of publications in recent years shows that the “tsunami” of the association between the microbiome and diabetes ranks among the top three (only after cancer and obesity) in the fields of diseases and gut microbiota research [13]. Gut microbiota contributes to metabolic health by producing some biologically active metabolites and leads to metabolic disorders due to the “dysbiosis deluge” [13]. Recent studies have shown that T2DM patients are characterized by gut microbiota dysbiosis, notably a reduction in short-chain fatty acid (SCFA)-producing bacteroides, and restored healthy gut microbiota composition by fecal microbiota transplantation effectively improved insulin resistance both in humans and mice with T2DM [14,15]. As the modulator of host metabolism and the immune system, dietary constituents are the pivotal determinant of gut microbiota community structure and function [16]. For instance, a high-fat diet (HFD) is suggested to decrease the abundance of SCFA-producing bacteroides, and a consequent deficiency in SCFAs (especially butyric acid) exacerbates T2DM [17]. Considering that fermentation of dietary fiber is the major source for SCFAs; therefore, its supplementation has been widely adopted in the nutritional therapy for T2DM [18]. Of particular importance was that partially hydrolyzed guar gum (PHGG), a water-soluble dietary fiber, was effective in enriching SCFA-producing bacteria (e.g., *Bifidobacterium* and *Ruminococcus*), which were depleted in T2DM, and increasing fecal SCFAs in both animals and humans [19]. However, previous studies have focused on intestinal diseases, while it remains unclear whether PHGG can improve T2DM and what the underlying mechanism of action is.

In this study, we first investigated the effects of LFD on disorders of glucose and lipid metabolism in T2DM mice. Moreover, to develop a more effective strategy based on LFD, we formulated a PHGG-containing diet and explored the effects of this combination on glucose metabolism disorders in T2DM mice. Finally, to explore the mechanism, 16S rRNA sequencing, untargeted metabolomics, and targeted metabolomics were performed to identify the key gut microbiota composition and fecal metabolites modulated by PHGG in T2DM mice.

## 2. Materials and Methods

### 2.1. Materials

Food-grade guar gum (mannose/galactose ratio = 1.67–1.70, galactomannan ≥ 90%, CAS No.: 9000-30-0) was provided by Qingzhou Ronmer Biology Technology Co., Ltd. (Qingzhou, China); its extraction method and physical-chemical properties have been described elsewhere [20]. LDL-C (low-density lipoprotein cholesterol), HDL-C, TC, TG, and insulin test kits were provided by Nanjing Jiancheng Bioengineering Institute (Nanjing, China). Primary antibody included anti-F4/80 (Abcam ab300421, Cambridge, UK). The RNA extraction kit was purchased from Vazyme Biotech Co., Ltd. (Nanjing, China). PrimeScript™ FAST RT Reagent Kit with gDNA Eraser (Takara RR092A, Takara Bio, Kusatsu, Japan) and TB Green^®^ Premix Ex Taq™ II FAST qPCR (Takara CN830A) were provided by Takara Biomedical Technology Co., Ltd. (Beijing, China). All of the other chemicals used in this study were of reagent grade and were purchased from a local reagent supplier.

### 2.2. Animals

Eight-week-old male C57BL/6J mice (*n* = 41) were purchased from Henan Scibes Biotechnology Co., Ltd. (Zhengzhou, China). Mice were housed in specific pathogen-free facilities at 22–24 °C under a 12 h light/12 h dark cycle. Food and water were provided ad libitum. All animals were treated in line with the “Guide for the Care and Use of Laboratory Animals” prepared by the National Academy of Sciences and published by the National Institutes of Health (NIH publication 86–23 revised 1985), and all animal procedures were supported by the Animal Care and Use Committee of Huazhong University of Science and Technology (approval code: No. 3017; date of ethical approval: 17 May 2022). 

According to a previous study [21], to establish the T2DM model, mice were fed with HFD (42% carbohydrate, 24% fat, and 24% protein, in mass) for 8 weeks, and subsequently administered streptozotocin (STZ, 50 mg/kg body weight) for five consecutive days. Fasting blood glucose (FBG) was measured after one week, and mice with FBG ≥ 11.1 mmol/L were defined as T2DM and used in further study. Age-matched control mice (*n* = 8) were fed a chow diet (CD, 54% carbohydrate, 4.5% fat, 20% protein, in mass). Potential confounders, such as the order of treatments and measurements, animal/cage location, operator, and instruments, were minimized by standardizing the timing of treatments and measurements for all animals and ensuring consistent environmental conditions across cages. Additionally, each group of cages was evenly distributed across different levels of the cage rack, rather than being concentrated only on certain levels.

For the LFD reversal study, T2DM mice were fed with HFD for 8 weeks, and then a part of the mice were switched to LFD for 4 weeks (DM-LFD, *n* = 9), whereas a group of T2DM mice were kept on HFD (DM-HFD, *n* = 8). For the PHGG intervention study, we formulated a PHGG-containing diet comprising 2.5% PHGG (PHGG-L, *n* = 8) or 7.5% PHGG (PHGG-H) based on HFD or LFD. Next, T2DM mice were exposed to a PHGG-containing diet for 12 weeks.

The number of mice in each group was determined with reference to previous studies on the relationship between gut microbiota and diabetes [22]. The study found that 6 mice per group were sufficient to detect the effects of gut microbiota on the alleviation of T2DM [22]. To achieve a statistical power of at least 80% for comparisons at a significance level of α = 0.05, considering that the mortality was 10%, the sample size was set at 8, which was in line with the “3R” (Replacement, Reduction, and Refinement) principle of animal ethics. Adequate power for one-way ANOVA analysis across three or four groups was provided, which complied with established international standards for rigor and ethical animal use in preclinical research. No specific pre-established inclusion or exclusion criteria were used, and all the mice remained alive throughout the entire experiment. The mouse keepers, data analysts, and sample testers were all unaware of the specific groupings, and the supervisor is aware of the grouping information. Breaking the blind was carried out after each stage of the experiment was completed.

### 2.3. Cell Culture and Treatment

The immortalized mouse hepatocyte cell line AML12 was cultured in DMEM/F12 supplemented with 10% fetal bovine serum, a mixture of insulin-transferrin-selenium (Sigma-Aldrich, I1884, St. Louis, MO, USA), 0.1 mM dexamethasone, 1% penicillin, and streptomycin. Palmitic acid (PA, Sigma-Aldrich, P0500) was completely dissolved in 0.1 M NaOH by heating at 90 °C, and then diluted in DMEM containing 5% (*w*/*v*) fatty acid-free bovine serum albumin (Yeasen, Shanghai, China) to 20  mM. Cells were treated with normal glucose (5.5 mM) and PA (200 μM) plus high glucose (HG, 30 mM) (PAHG) with or without the addition of PHGG (1 mg/mL) for 24 h.

### 2.4. Intraperitoneal Glucose Tolerance Test

After fasting for 12 h, mice were injected with glucose (2 g/kg body weight) intraperitoneally. Blood glucose was measured by using glucose test strips and a glucometer (Yuwell, Danyang, China) every 30 min from 0 to 120 min post-injection, and the area under the curve (AUC) was calculated to evaluate glucose tolerance.

### 2.5. 16S rRNA Sequencing

Briefly, total genomic DNA from feces was extracted using the CTAB method. The V3-V4 region of the 16S rRNA gene was amplified with primers 341F: 5′-CCTACGGGNGGCWGCAG-3′, and 805R: 5′-GACTACHVGGGTATCTAATCC-3′. All PCR reactions were carried out with Phusion^®^ High-Fidelity PCR Master Mix (New England Biolabs, Ipswich, MA, USA). PCR products were purified with a Qiagen Gel Extraction Kit (Qiagen, Hilden, Germany). Sequencing libraries were generated using the TruSeq^®^ DNA PCR-Free Sample Preparation Kit (Illumina, San Diego, CA, USA).

### 2.6. Fecal Short-Chain Fatty Acids

Fecal samples (20 mg) were homogenized in 1 mL of 0.5% (*v*/*v*) phosphoric acid using a steel bead. After centrifugation (12,000 rpm, 10 min, 4 °C), 0.1 mL supernatant was mixed with 0.5 mL MTBE (containing internal standard). Post-centrifugation (same conditions), the Agilent 7890B gas chromatograph coupled to a 7000D mass spectrometer with a DB-FFAP column (30 m length × 0.25 mm i.d. × 0.25 μm film thickness, J&W Scientific, Folsom, CA, USA) was employed for GC-MS/MS analysis of SCFAs in the supernatant.

### 2.7. Hematoxylin and Eosin Staining

Liver tissues were embedded in paraffin wax, cut into 5-μm sections, and mounted on glass slides. Sections were dewaxed in xylene and hydrated in descending ethanol solutions. Sections were stained with hematoxylin for 5 min and 1% eosin for 5 min, and mounted on stained and dried slides with xylene. The stained sections were observed under a digital image microscope (MshOt, Guangzhou, China).

### 2.8. Oil Red O Staining

Frozen liver tissues were embedded in optimal cutting temperature compound, sectioned to 5-μm thickness with a freezing microtome. Sections were mounted on glass slides and fixed in 4% formalin for 15 min. Sections were stained with 60% Oil Red O working solutions for 15 min and hematoxylin for 2 min. Cover slips were mounted on the sections using glycerol gelatin. The stained sections were observed under a digital image microscope (MshOt, Guangzhou, China).

### 2.9. Fasting Blood Glucose Measurement

Mice were fasted overnight (12 h), anesthetized, and blood was collected, and fasting blood glucose levels were measured by using glucose test strips and a glucometer (Yuwell, Danyang, China).

### 2.10. Serum Detection

The serum of mice was collected after fasting for 12 h. Serum TC, TG, HDL-C, and LDL-C were measured by using biochemical kits (Nanjing Jiancheng Bioengineering Institute, Nanjing, China) according to the manufacturer’s instructions.

### 2.11. RNA Extraction and Real-Time PCR Analysis

As previously studied [23], briefly, total RNA was isolated from liver tissues and cells using an RNA extraction kit (Takara Bio, Kusatsu, Japan). For reverse transcription, cDNA was synthesized from RNA samples with the PrimeScript™ FAST RT Reagent Kit (Takara Bio, Kusatsu, Japan) with gDNA Eraser. Quantitative real-time PCR was performed with TB Green^®^ Premix Ex Taq™ II FAST qPCR using the Applied Biosystems 7500 Real-time PCR System (Waltham, MA, USA) according to the manufacturer’s instructions. Gene expression levels were calculated after normalization to the housekeeping gene GAPDH. The primer sequences are provided in Table 1.

### 2.12. Statistical Analysis

Data are presented as mean ± SD. Statistical significance between two groups was calculated using an unpaired, two-tailed *t*-test or Mann–Whitney test. Data from more than two groups was compared using a one-way ANOVA followed by Dunnett’s multiple comparisons test or a Kruskal–Wallis test with Dunn’s multiple comparisons test. The Spearman correlation was used to establish the association between the two variables. *p* < 0.05 was considered statistically significant. All data were analyzed based on at least three replicate experiments and were analyzed with the GraphPad Prism 9.0 program (GraphPad Software).

## 3. Results

### 3.1. Reducing Saturated Fat Intake Improved Dyslipidemia but Not Glucose Metabolism Disorders in T2DM Mice

**Figure 1:**
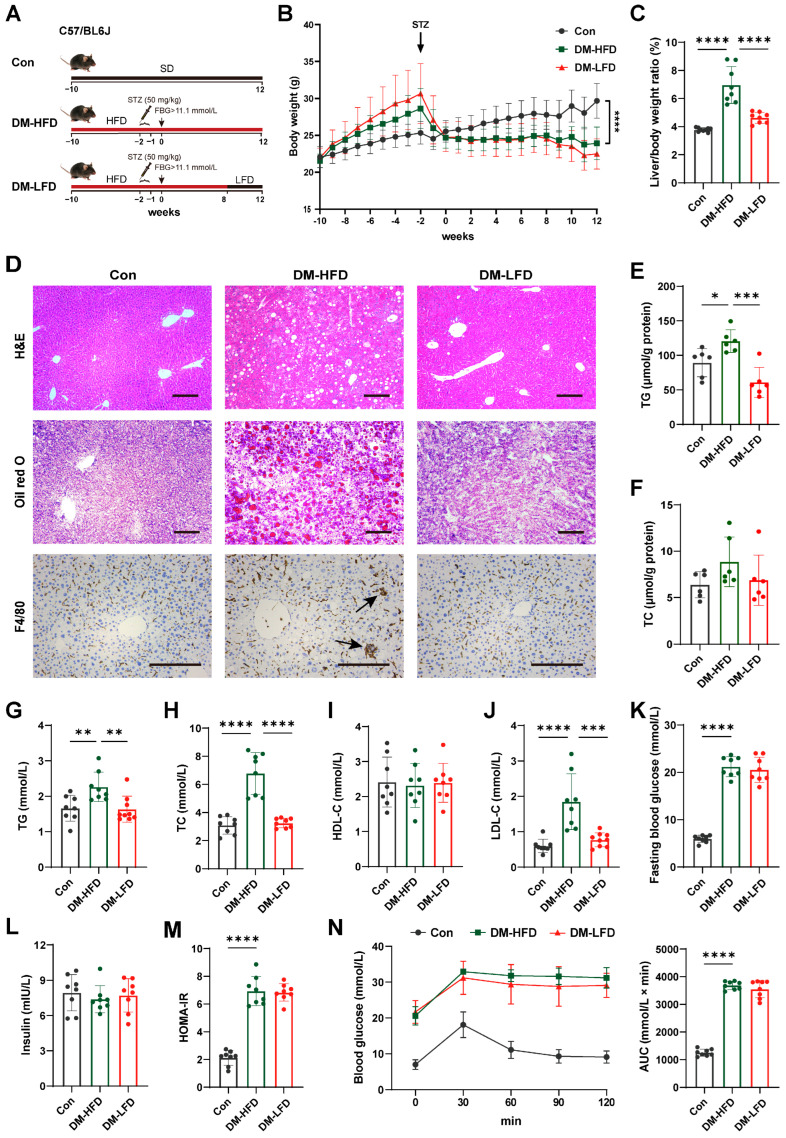
LFD ameliorated lipid metabolism disorders but not glucose metabolism disorders in T2DM mice. (**A**) Study design of the LFD treatment. Con: healthy mice fed with CD; DM-HFD: T2DM mice fed with HFD for 12 weeks; DM-LFD: T2DM mice fed with HFD for 8 weeks, then switched to LFD for 4 weeks. (**B**) Body weight (*n* = 8). (**C**) Liver-to-body weight ratio (*n* = 8). (**D**) Representative images of H&E staining, Oil Red O staining, and F4/80 immunohistochemical staining of liver sections (*n* = 6). Scale bar, 50 µm. The arrow indicates the positive area. (**E**,**F**) The TG (*n* = 6) (**E**) and TC (*n* = 6) (**F**) levels of the livers. (**G**–**L**) Serum TG (*n* = 8–9) (**G**), TC (*n* = 8) (**H**), HDL-C (*n* = 8) (**I**), LDL-C (*n* = 8–9) (**J**), FBG (*n* = 8) (**K**), and fasting insulin (*n* = 8) (**L**). (**M**) HOMA-IR (*n* = 8). (**N**) IPGTT and AUC for glucose between 0 and 120 min (*n* = 8). Values are presented as means ± SD. Statistical analysis was performed using one-way ANOVA with Dunnett’s multiple comparisons test. * *p* < 0.05, ** *p* < 0.01, *** *p* < 0.001, **** *p* < 0.0001.

HFD induced liver lipid deposition, which caused the defects of hepatic insulin resistance and β-cell dysfunction in T2DM [24]. To observe the effects after the reduction in saturated fatty acid intake, an LFD intervention in T2DM mice was designed. As shown in Figure 1A, the T2DM model was first established, then one group of the T2DM mice was fed with an HFD for 8 weeks and then switched to an LFD for 4 weeks (DM-LFD), and another group of T2DM mice was still kept on HFD (DM-HFD). Compared to DM-HFD mice, less saturated fatty acid intake allowed the DM-LFD mice to have a lower liver-to-body weight ratio, although there was no significant decrease in body weight (Figure 1B,C). Notably, 4-week LFD administration was sufficient to alleviate HFD-induced hepatic steatosis, hepatocyte ballooning, and lobular inflammation, as evidenced by H&E staining, Oil Red O staining, and F4/80 immunohistochemical staining (Figure 1D). Moreover, LFD significantly decreased the hepatic TG (by 50%, *p* < 0.001), as well as the decreasing trend of hepatic TC (Figure 1E,F). In addition, LFD significantly reduced serum TG from 2.26 ± 0.39 mM to 1.64 ± 0.35 mM (by 28%, *p* < 0.01), TC from 6.78 ± 1.39 mM to 3.26 ± 0.31 mM (by 52%, *p* < 0.0001), and LDL-C from 1.85 ± 0.73 mM to 0.77 ± 0.19 mM (by 58%, *p* < 0.001) in T2DM mice (Figure 1G–J). However, neither FBG nor HOMA-IR was improved by LFD administration (Figure 1K–M). Furthermore, it was shown that LFD feeding failed to improve the impaired glucose tolerance (Figure 1N). Together, LFD alone could ameliorate lipid metabolism disorders, but failed to reverse hyperglycemia, insulin resistance, and impaired glucose tolerance in T2DM mice.

### 3.2. PHGG Improved Hyperglycemia, Insulin Resistance, and Decreased Glucose Tolerance in T2DM Mice

**Figure 2:**
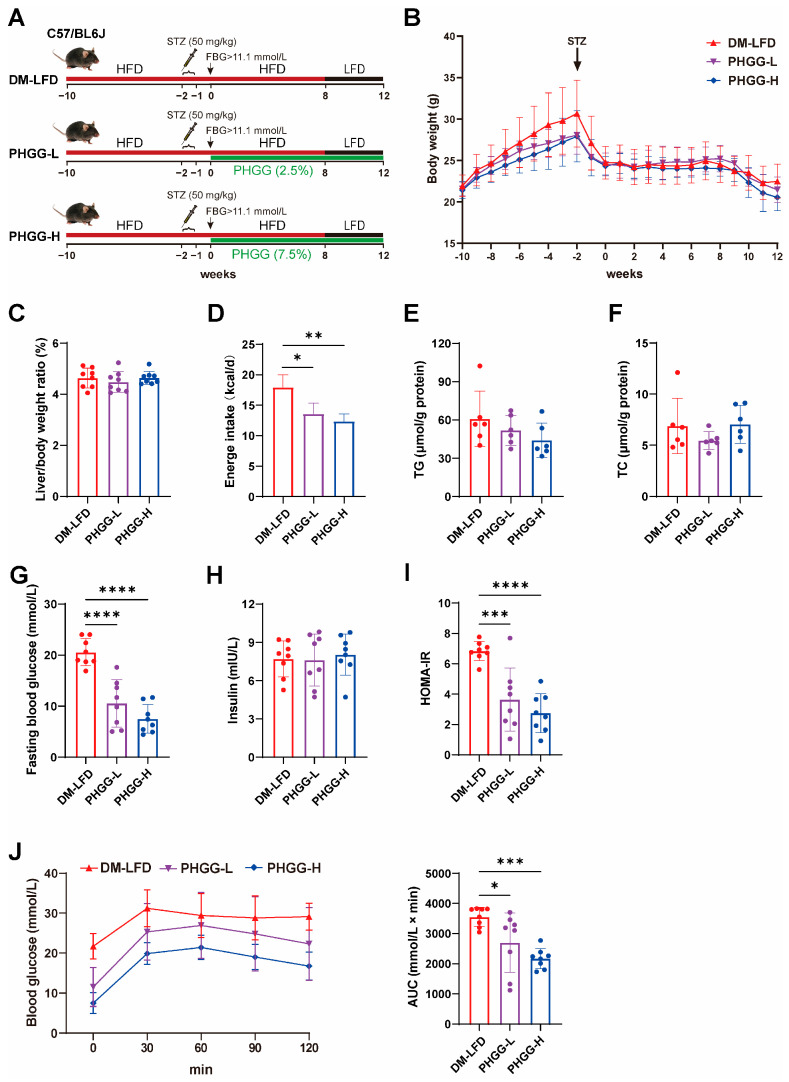
PHGG ameliorated hyperglycemia, insulin resistance, and decreased glucose tolerance in T2DM mice. (**A**) Study design of PHGG treatment in DM-LFD mice. T2DM mice were fed with a PHGG-containing diet (2.5%, PHGG-L; 7.5%, PHGG-H) for 12 weeks, and DM-LFD was used as a control group. (**B**) Body weight (*n* = 8). (**C**) Liver-to-body weight ratio (*n* = 8). (**D**) Daily energy intake from 8 to 12 weeks (*n* = 8). (**E**,**F**) The TG (*n* = 6) (**E**) and TC (*n* = 6) (**F**) levels of the livers. (**G**,**H**) FBG (*n* = 8) (**G**) and insulin (*n* = 8) (**H**) in serum (*n* = 8). (**I**) HOMA-IR. (**J**) IPGTT and AUC for glucose between 0 and 120 min (*n* = 8). Values are presented as means ± SD. Statistical analysis was performed using ANOVA with the Dunnett multiple comparisons test. * *p* < 0.05, ** *p* < 0.01, *** *p* < 0.001, **** *p* < 0.0001.

Dietary fiber has been reported to improve hyperglycemia and insulin resistance in patients with T2DM [18]. To test whether LFD combined with PHGG supplementation could reverse glucose metabolic disorders in T2DM mice, we formulated the diets comprising 2.5% or 7.5% PHGG, respectively. T2DM mice were treated with the PHGG-based diets for 12 weeks (PHGG-L, PHGG-H), and DM-LFD was the control group (Figure 2A). Compared with the DM-LFD group, mice in the PHGG-L and PHGG-H groups had similar body weight and liver-to-body weight ratio, but significantly reduced hyperphagia, that is, lower energy intake (Figure 2B–D). Consistent with the DM-LFD group, PHGG combined with LFD also displayed a favorable lipid-lowering effect (Figure 2E,F). Importantly, PHGG-L and PHGG-H significantly reduced FBG from 20.55 ± 2.49 mM to 10.53 ± 4.33 mM and 7.49 ± 2.64 mM, respectively (by 49% and 64%, *p* < 0.0001), HOMA-IR from 6.84 ± 0.59 to 3.64 ± 1.94 and 2.75 ± 1.20, respectively (by 47% and 60%, *p* < 0.001), and improved glucose tolerance (by 24% and 39%, *p* < 0.05) in a dose-effect manner (Figure 2G–J).

### 3.3. PHGG Modulated Gut Microbiota Composition in T2DM Mice

**Figure 3:**
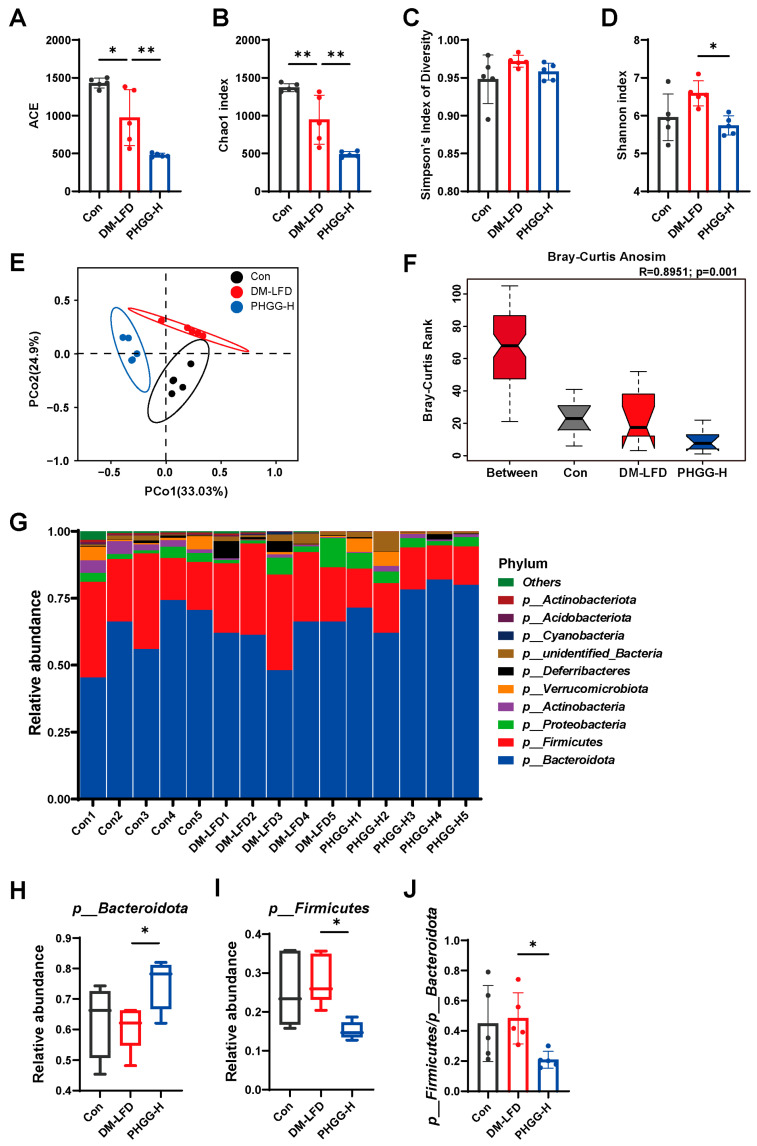
PHGG modulated gut microbiota composition in T2DM mice. The 16S rRNA gene sequence amplicon datasets displayed the gut microbiota of healthy control mice and T2DM mice treated with LFD (DM-LFD) or LFD plus 7.5% PHGG (PHGG-H). (**A**–**D**) Alpha diversity based on ACE, Chao1, the Shannon index, and Simpson’s index of diversity (*n* = 5). Data are shown as mean ± SD. Statistical analysis was performed using ANOVA with Dunnett’s multiple comparisons test. (**E**,**F**) PCoA and Anosim by Bray–Curtis in all samples (*n* = 5). The circles represent the 95% confidence intervals for each group. (**G**) Relative abundance of the top 10 most abundant gut microbiota bacterial phylum (*n* = 5). (**H**,**I**) Relative abundance of Bacteroidetes and Firmicutes (*n* = 5). Data are shown as median [IQR]. Statistical analysis was performed using the Kruskal–Wallis test with a Dunn’s multiple comparisons test. (**J**) The ratio of the relative abundance of Firmicutes to Bacteroidetes (*n* = 5). IQR, interquartile range. * *p* < 0.05, ** *p* < 0.01.

Considering PHGG-H was more effective than PHGG-L in the improvement of T2DM, mice in the PHGG-H group were selected for further study. Therefore, we performed 16S rRNA sequencing analysis to investigate the differences in gut microbiota composition derived from the Con, DM-LFD, and PHGG-H mice. We measured alpha diversity using ACE, Chao1, the Shannon index, and Simpson’s index of diversity (Figure 3A–D). Then, beta diversity analysis of principal coordinates analysis (PCoA) was performed using Bray–Curtis (Figure 3E). It was shown that the gut microbiota from different groups were largely separated, suggesting that these community structures were distinct among the groups. Consistently, a statistically significant separation among the three groups was evidenced by analysis of similarities (Anosim) (*p* = 0.001, Figure 3F). The relative abundances of the top 10 most abundant gut microbiota bacterial phylum were displayed in Figure 3G. Compared with the DM-LFD group, PHGG treatment significantly decreased the Firmicutes/Bacteroidetes ratio at the phylum level, which was due to the increase in Bacteroidetes and the reduction in Firmicutes (Figure 3H–J).

### 3.4. Short-Chain Fatty Acid-Producing Bacteria Were Enriched by PHGG in T2DM Mice

**Figure 4:**
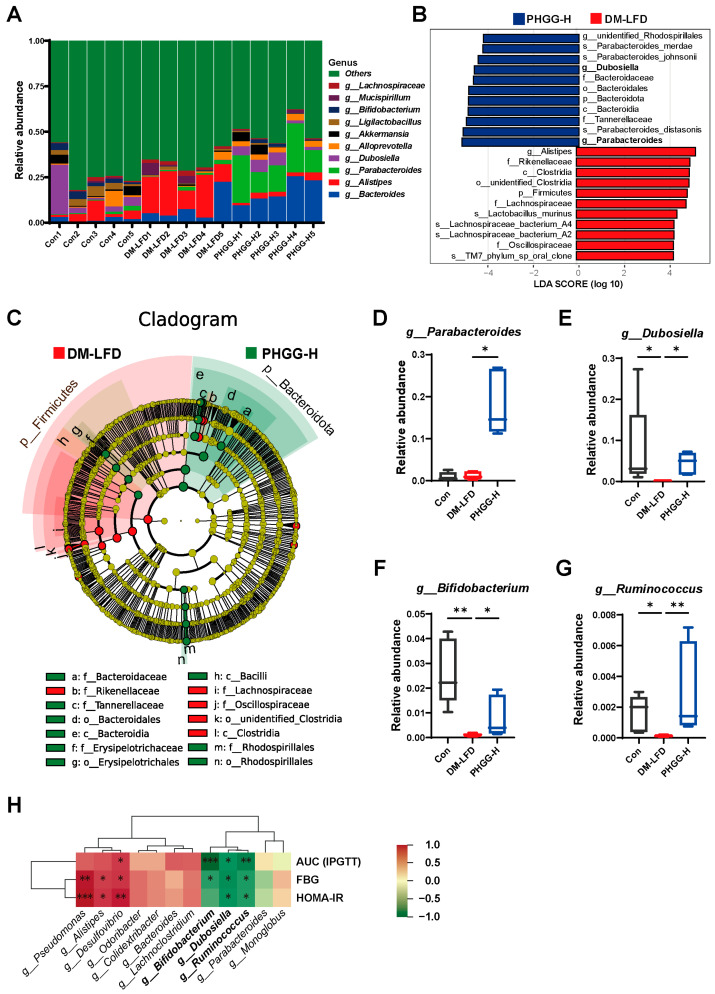
SCFA-producing bacteria were enriched by PHGG in T2DM mice. (**A**) Relative abundance of the top 10 most abundant gut microbiota bacterial genera (*n* = 5). (**B**) Analysis of the differences in the gut microbiota by LEfSe (LDA > 4) (*n* = 5). (**C**) Taxonomic cladogram from LEfSe, depicting taxonomic association from microbiome communities between DM-LFD and PHGG-H groups (*n* = 5). The microbial taxa that play important roles in the DM-LFD group and the PHGG-H group are marked in red and green respectively. Each node represents a specific taxonomic type. Yellow nodes denote the taxonomic features that are not significantly differentiated. Red nodes represent the taxonomic types that are more abundant in the DM-LFD group than in the PHGG-H group, while the green nodes represent the taxonomic types more abundant in the PHGG-H group. (**D**–**G**) Relative abundance of bacterial genera *Parabacteroides*, *Dubosiella*, *Bifidobacterium*, and *Ruminococcus* (*n* = 5). Statistical analysis was performed using the Kruskal–Wallis test with a Dunn’s multiple comparisons test. (**H**) Spearman’s correlation analysis of the 12 identified genera and T2DM traits (*n* = 5). Data are presented as median [IQR]. IQR, interquartile range. * *p* < 0.05, ** *p* < 0.01, *** *p* < 0.001.

At the genus level, the dominant bacterial genus changed greatly, and the relative abundances of the top 10 most abundant bacterial genera are shown in Figure 4A. Taxonomic composition differences between the DM-LFD and PHGG groups were evaluated by Linear Discriminant Analysis (LDA) effect size (LEfSe) (LDA > 4) (Figure 4B). Notably, the genera *Parabacteroides* and *Dubosiella* were greatly enriched by PHGG-H (LDA = 5.14 and 4.59, respectively), and the relative abundances were displayed in Figure 4C,D. In addition, the relative abundances of genus *Bifidobacterium* and *Ruminococcus* were depleted in DM-LFD mice but significantly enriched after PHGG-H treatment (Figure 4F,G). Furthermore, the correlation between the 12 bacterial genera altered by PHGG-H and the traits of T2DM was further analyzed (Figure 4H). Spearman’s correlation analysis showed that the relative abundances of genus *Dubosiella*, *Bifidobacterium*, and *Ruminococcus* were negatively correlated with FBG, HOMA-IR, and AUC (IPGTT). These findings indicated that PHGG might ameliorate T2DM by modulating gut microbiota composition.

### 3.5. PHGG Treatment Altered Fecal Metabolic Profiles in T2DM Mice

**Figure 5:**
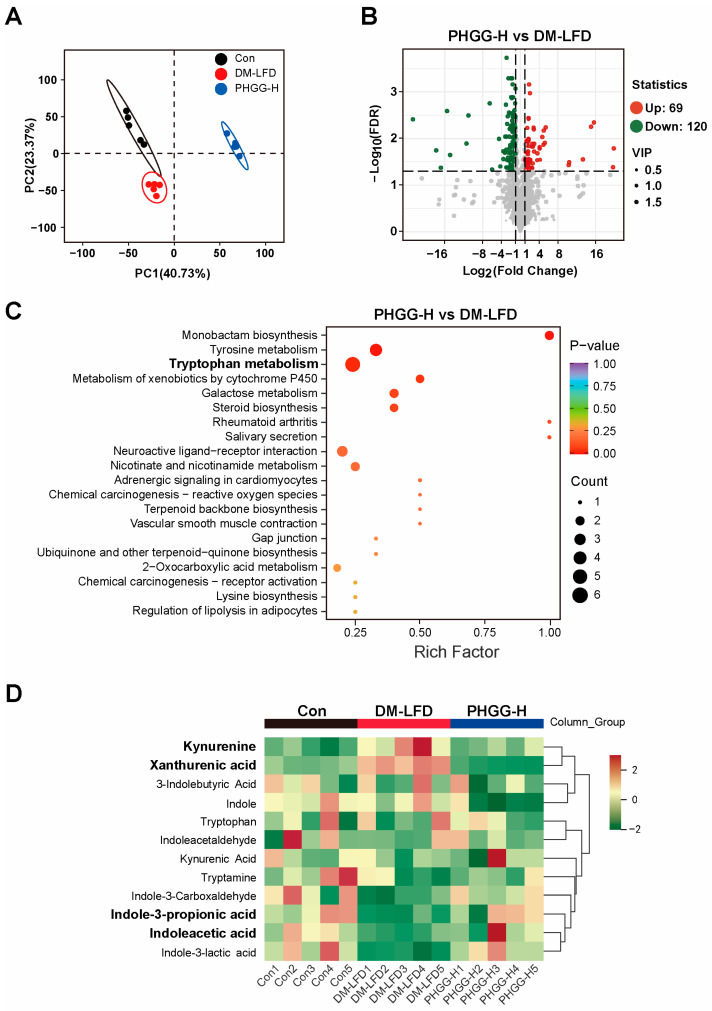
PHGG treatment altered metabolic profiles in T2DM mice’s stool samples. Untargeted metabolomics was conducted with mice’s stool samples in the Con, DM-LFD, and PHGG-H groups. (**A**) PCA of fecal metabolites (*n* = 5). The circles represent the 95% confidence intervals for each group. (**B**) Volcano plots showed altered fecal metabolites in the PHGG-H group compared to the DM-LFD group (*n* = 5). (**C**) KEGG enrichment analysis (*n* = 5). (**D**) Clustering heatmap of tryptophan metabolites of mice’s stool samples from the Con, DM-LFD, and PHGG-H groups (*n* = 5).

To profile the metabolic changes, untargeted metabolomics was conducted to analyze the mice’s stool samples in the Con, DM-LFD, and PHGG-H groups. A total of 1837 metabolites were identified, and principal component analysis (PCA) revealed that the metabolite profiles of the PHGG-H group were clearly separated from those of the DM-LFD group, indicating that PHGG supplementation altered gut metabolite profiles by gut microbiota in T2DM mice (Figure 5A). Compared with the DM-LFD group, PHGG-H supplementation significantly upregulated 69 metabolites and downregulated 120 metabolites (VIP > 1, FDR < 0.05, and FC > 2, Figure 5B). To gain further insight into the potential functions of these metabolites, KEGG enrichment analysis was performed. As shown in Figure 5C, the differential metabolites were mainly enriched in tryptophan metabolism, tyrosine metabolism, metabolism of xenobiotics by cytochrome P450, galactose biosynthesis, and the steroid biosynthesis pathway. Notably, tryptophan is an essential amino acid that plays a critical role in T2DM and can be catabolized to various metabolites through host kynurenine and microbial indole pathways [25,26]. Fecal tryptophan metabolite levels among the Con, DM-LFD, and PHGG-H groups are shown in Figure 5D. Indole derivatives (indoleacetic acid and indole-3-propionic acid), which were reported to be associated with decreased risk of T2DM [27], were enriched in stool samples from PHGG-H mice. Meanwhile, the kynurenine-pathway metabolites (kynurenine and xanthurenic acid), which were associated with increased risk of T2DM [28], were depleted after PHGG treatment.

### 3.6. PHGG Restored Fecal Short-Chain Fatty Acid Production in T2DM Mice

**Figure 6:**
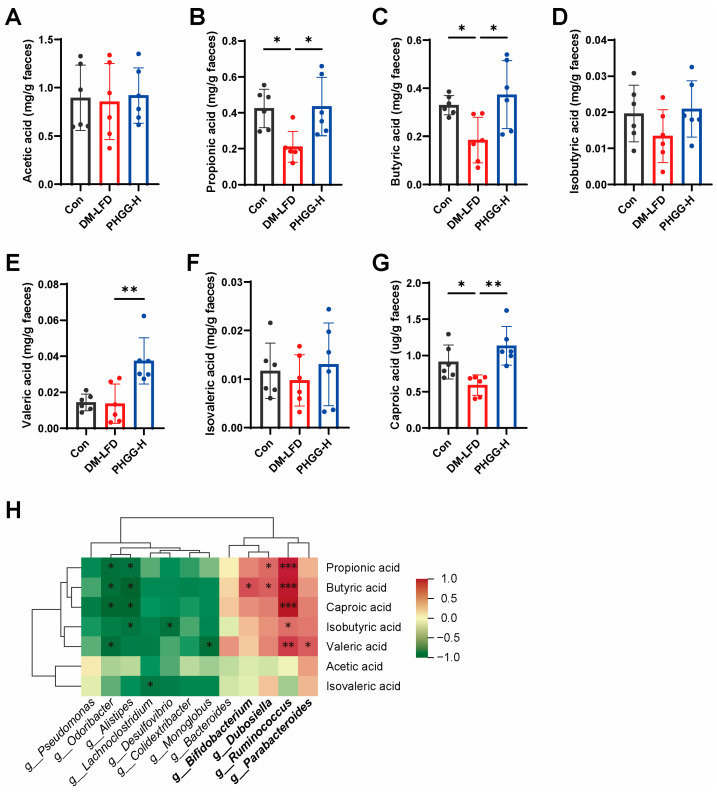
PHGG restored SCFA production in T2DM mice. (**A**–**G**) Acetic acid (*n* = 6) (**A**), propionic acid (*n* = 6) (**B**), butyric acid (*n* = 6) (**C**), isobutyric acid (*n* = 6) (**D**), valeric acid (*n* = 6) (**E**), isovaleric acid (*n* = 6) (**F**), and caproic acid (*n* = 6) (**G**) in mice’s stool samples from the Con, DM-LFD, and PHGG-H groups. (**H**) Spearman’s correlation analysis between fecal SCFAs and the relative abundance of 12 identified genera (*n* = 5). * *p* < 0.05, ** *p* < 0.01, *** *p* < 0.001.

SCFA production deficiency is associated with an increased risk of T2DM, while gut microbiota is beneficial to humans via producing SCFAs from carbohydrate fermentation [29]. Therefore, we measured fecal SCFAs in mice from the groups of the Con, DM-LFD, and PHGG-H. Compared with non-diabetic mice, fecal SCFAs were greatly decreased in T2DM mice, including propionic acid, butyric acid, and caproic acid, which were rescued by PHGG treatment (Figure 6A–G). Given that SCFA production is dependent on gut microbiota, we further performed Spearman’s correlation analysis to explore the association between fecal SCFAs and 12 bacterial genera altered by PHGG (Figure 6H). The results showed that fecal SCFAs, especially propionic acid and butyric acid, were positively associated with the relative abundance of the genera *Dubosiella* and *Ruminococcus*, which were enriched by PHGG. Taken together, PHGG treatment might elevate fecal SCFAs by restoring SCFA-producing bacteria in T2DM mice.

### 3.7. PHGG Improved Liver Gluconeogenesis in T2DM Mice

**Figure 7:**
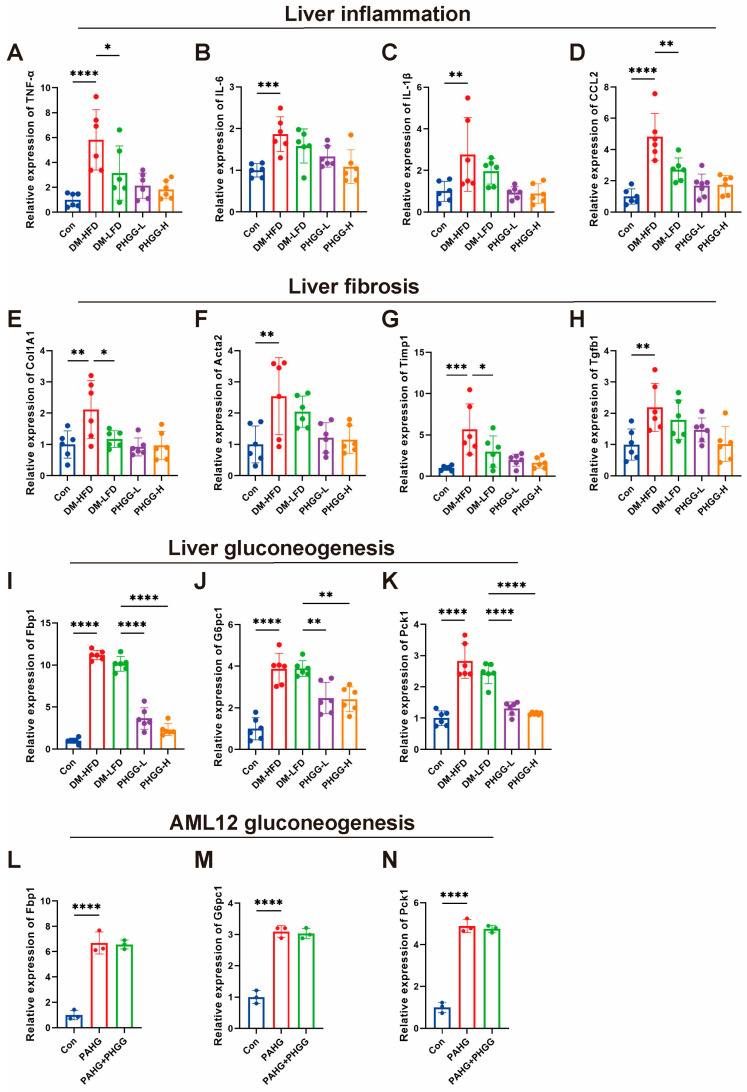
PHGG improved liver gluconeogenesis in T2DM mice. (**A**–**D**) The relative expression of mRNA related to inflammation (*TNF-α*, *IL-6*, *IL-1β*, and *CCL2*) in the livers of T2DM mice (*n* = 6). (**E**–**H**) The relative expression of mRNA related to fibrosis (*Col1A1*, *Acta2*, *Timp1*, and *Tgfb1*) in the livers of T2DM mice (*n* = 6). (**I**–**K**) The relative expression of mRNA related to gluconeogenesis (*Fbp1*, *G6pc1*, and *Pck1*) in the livers of T2DM mice (*n* = 6). (**L**–**N**) The relative expression of mRNA related to gluconeogenesis (*Fbp1*, *G6pc1*, and *Pck1*) in the AML12 hepatocyte line treated with PA (200 μM) plus high glucose (HG, 30 mM) in the presence or absence of PHGG (1 mg/mL). PAHG, palmitic acid plus high glucose. * *p* < 0.05, ** *p* < 0.01, *** *p* < 0.001, **** *p* < 0.001.

The key genes related to liver inflammation, fibrosis, and gluconeogenesis were detected to explore the potential mechanism by which PHGG improved T2DM through the gut microbiota. As shown in Figure 7A–D, the expression of *TNF-α*, *IL-6*, *IL-1β*, and *CCL2* was significantly increased in the livers of T2DM mice, and was observably decreased in the DM-LFD group, which was comparable to the inflammatory levels after intervention with PHGG. Similarly, the levels of mRNA related to liver fibrosis (*Col1A1*, *Acta2*, *Timp1*, and *Tgfb1*) were significantly increased in the DM group, which were significantly reduced by the LFD treatment. After PHGG intervention, although there was no difference with the DM-LFD group, a further decreasing trend was observed (Figure 7E–H). Importantly, the expression of *Fbp1*, *G6pc1*, and *Pck1* was markedly increased in the livers of T2DM mice, but was not reduced by LFD administration, which was significantly decreased with PHGG treatment in a dose-dependent manner. In vitro, the expression of mRNAs related to gluconeogenesis was significantly induced in hepatocytes treated with high glucose and high lipid, but was not reduced by the direct treatment of PHGG, suggesting that PHGG might improve the liver’s gluconeogenesis by regulating the intestinal flora, rather than directly acting on liver cells (Figure 7L–N).

## 4. Discussion

Despite the widespread application of the LFD in managing T2DM, its clinical utility is constrained by limited efficacy and poor long-term adherence. In this study, we found that the combination of PHGG with LFD yields superior therapeutic outcomes compared to LFD alone in alleviating T2DM. Firstly, PHGG supplementation markedly ameliorated T2DM, evidenced by reduced FBG, lowered HOMA-IR, and improved glucose tolerance. Furthermore, PHGG supplementation modulated gut microbiota composition, specifically enriching SCFA-producing bacteria, including the genera *Parabacteroides*, *Dubosiella*, *Bifidobacterium*, and *Ruminococcus*. Finally, PHGG significantly enhanced fecal tryptophan and tyrosine metabolism while promoting the production of propionic acid and butyric acid. These findings indicated that PHGG is a promising dietary supplement to cooperate with LFD for T2DM management, which provides a reference for the treatment of diseases that require limiting energy intake.

The twin cycle hypothesis supported that energy restriction, such as LFD, would reduce hepatic steatosis, normalize the liver’s insulin sensitivity, and liver glucose production [4]. Consistent with previous RCTs, our study confirmed that a 4-week LFD management effectively reversed lipid metabolism disorders by reducing TC, TG, and LDL-C levels [9,30]. Taylor et al. reported that the decrease in liver lipid content could alleviate T2DM, but the remission must be dependent on the capacity of β-cell recovery. Insulin resistance is a core pathological feature of T2DM, and it is extremely difficult to restore through lipid-lowering and weight loss alone [31]. Similarly, normalization of liver lipid content and serum lipid metabolism was insufficient to improve liver insulin resistance, hyperglycemia, and decreased glucose tolerance in our present study.

Recent studies have shown that T2DM patients are characterized by gut microbiota dysbiosis, especially a decrease in SCFA-producing bacteria, a perturbation that dietary fiber intervention has been shown to reverse [32]. Among approximately six dietary fiber sources designated by the Food and Drug Administration of Korea, PHGG is registered as a functional ingredient [33]. Naito and colleagues also found that a high-fiber diet (5% PHGG) treatment for 20 weeks obviously decreased the AUC of OGTT and HOMA-IR and inhibited the development of diabetic complications in rats [34]. Importantly, a single-arm, pre-post comparison pilot clinical trial showed that the levels of FBG and HbA1c in the participants who consumed 12.5 g of PHGG exhibited a reduction from 106.68 ± 21.56 mg/dL to 101.54 ± 16.79 mg/dL, and 6.01% ± 0.77% to 5.86% ± 0.49%, respectively [32]. Vuorinen-Markkola et al. reported that patients with diabetes receiving 20 g daily of PHGG for six weeks showed 19.5% and 7.2% reductions in FBG and HbA1c, respectively [35]. Similar benefits were observed in healthy individuals consuming 30 g daily, with a 6.25% reduction in FBG and a 7.3% reduction in blood cholesterol [36]. Consistent with the results above, we also observed a significant improvement in HOMA-IR, FBG, and the IPGTT after PHGG (7.5%) administration in T2DM mice.

Tryptophan is an essential amino acid that plays a critical role in human health and disease, as it is the sole source for the kynurenine pathway, involved in immune activation and inflammation regulation [37], and has been associated with obesity and insulin resistance [27,38]. Aligning with previous studies, we found that the tryptophan metabolism pathway was strongly changed; that is, kynurenine and xanthurenic acid were elevated, but microbiota-derived metabolites, indoleacetic acid and indole-3-propionic acid, were decreased in the stool samples from mice with T2DM. However, the abnormal changes in these metabolites were restored by PHGG treatment. In line with this, Qi and colleagues also observed that kynurenine and xanthurenic acid positively correlated with T2DM, whereas indole-3-propionic acid exhibited a negative correlation, and dietary fiber intake promoted a more favorable tryptophan metabolite profile [27]. The restoration of indoleacetic acid and indole-3-propionic acid by PHGG is significant, as these metabolites are known to activate aryl-hydrocarbon receptors and suppress inflammation, a key pathological process in T2DM [39].

SCFAs were produced by the SCFA-producing bacteria. It was reported that PHGG restored the relative abundance of *Dubosiella*, a genus depleted in T2DM and colitis, had a negative association with FBG, HOMA-IR, and inflammatory cytokines [40,41], enriched *Bifidobacterium* and *Ruminococcus*, increased fecal SCFAs, and alleviated intestinal inflammation and diarrhea [19,42,43]. SCFAs, such as propionic acid, butyric acid, were closely related to the onset or progression of T2DM, which increased insulin sensitivity via influencing metabolic pathways and receptor-mediated mechanisms at various tissue and organ sites [44]. Butyrate-producing bacteria were universally decreased in patients with T2DM as compared with healthy controls [45]. Conversely, butyrate and indole-3-propionic acid (the combination of propionic acid and indole ring) supplements were associated with improved insulin sensitivity, FBG, and HbA1c and decreased complications [46,47]. Mechanistically, the intermediates of propionic acid and butyrate may suppress hepatic gluconeogenesis, thereby improving glycemic control in T2DM [48]. Similarly, we also found that SCFAs, such as propionic acid, butyric acid, and caproic acid, were decreased in the stool samples of T2DM mice, but elevated by PHGG administration in this study. Given that, PHGG might be a promising functional food additive incorporated into porridge, soup, beverages, ice cream, yogurt, bread, and jam to enhance the viscosity and satiety of the food for T2DM patients. However, these findings are derived from animal studies with T2DM, and the causal relationship remains to be fully elucidated. Therefore, its hypoglycemic effect and protective mechanism necessitate further validation in both human clinical trials and additional animal studies.

## 5. Conclusions

In summary, we found that PHGG supplementation might effectively enhance the therapeutic efficacy of LFD in T2DM. Mechanistically, PHGG may exert its anti-diabetic effects by modulating gut microbiota composition, particularly enriching SCFA-producing bacteria (e.g., *Parabacteroides*, *Dubosiella*, *Bifidobacterium*, and *Ruminococcus*), thereby promoting propionate and butyrate production. These findings suggest that PHGG might be a promising prebiotic to ameliorate T2DM via modulating gut microbiota and its metabolites, but the underlying mechanisms still require further exploration.

## Figures and Tables

**Table 1:** Primers for qPCR.

Gene	Forward Primer	Reverse Primer
GAPDH	GGCAAATTCAACGGCACAGT	CTCGTGGTTCACACCCATCA
TNF-α	GATCGGTCCCCAAAGGGATG	TTTGCTACGACGTGGGCTAC
IL-6	CCCCAATTTCCAATGCTCTCC	CGCACTAGGTTTGCCGAGTA
IL-1β	TGCCACCTTTTGACAGTGATG	TGATGTGCTGCTGCGAGATT
CCL2	CACTCACCTGCTGCTACTCA	GGTCAGCACAGACCTCTCTC
Col1a1	CGTAGCCTACATGGACCAGC	CGATGACTGTCTTGCCCCAA
Acta2	CCACCATGTACCCAGGCATT	GAAGGTAGACAGCGAAGCCA
Timp1	ATCAGTGCCTGCAGCTTCTT	TCTGGTAGTCCTCAGAGCCC
Tgfb1	ACTGGAGTTGTACGGCAGTG	GGGGCTGATCCCGTTGATTT
Fbp1	GGCTCATCCAACATTGAC	CTCAGAAGGCTCATCAGT
G6pc1	CAAGTGAATTACCAAGAC	GGAAAGAGGACATAGAAG
Pck1	TTGCCTGGATGAAGTTTG	GTTGGTGAAGATGGTGTT

## Data Availability

The original contributions presented in this study are included in the article. Further inquiries can be directed to the corresponding authors.

## Abbreviations

The following abbreviations are used in this manuscript:

Anosim	Analysis of similarities
AUC	Area under the curve
DiRECT	Diabetes Remission Clinical Trial
FBG	Fasting blood glucose
GC-MS	Gas chromatography-mass spectrometry
HDL-C	High-density lipoprotein cholesterol
HFD	High-fat diet
HG	High glucose
HOMA-IR	Homeostatic model assessment-insulin resistance
IPGTT	Intraperitoneal glucose tolerance test
IQR	Interquartile range
KEGG	Kyoto Encyclopedia of Genes and Genomes
LDL-C	Low-density lipoprotein cholesterol
LDA	Linear Discriminant Analysis
LFD	Low-fat diet
PA	Palmitic acid
PAHG	Palmitic acid plus high glucose
PCoA	Principal coordinates analysis
PHGG	Partially hydrolyzed guar gum
SCFAs	Short-chain fatty acids
CD	Chow diet
STZ	Streptozotocin
T2DM	Type 2 diabetes mellitus
TC	Total cholesterol
TG	Triglycerides
